# Dynamic Transcriptome Changes Related to Oil Accumulation in Developing Soybean Seeds

**DOI:** 10.3390/ijms20092202

**Published:** 2019-05-05

**Authors:** Songnan Yang, Long Miao, Jianbo He, Kai Zhang, Yan Li, Junyi Gai

**Affiliations:** National Key Laboratory of Crop Genetics and Germplasm Enhancement, Key Laboratory for Biology and Genetic Improvement of Soybean (General, Ministry of Agriculture), National Center for Soybean Improvement, Jiangsu Collaborative Innovation Center for Modern Crop Production, Nanjing Agricultural University, Nanjing 210095, China; ysn785620774@126.com (S.Y.); 2016201034@njau.edu.cn (L.M.); hjbreg@163.com (J.H.); zkadrian@163.com (K.Z.)

**Keywords:** soybean, Seed development, RNA-Seq, gene co-expression network analysis, oil

## Abstract

Soybean is one of the most important oil crops in the world. Revealing the molecular basis and exploring key candidate genes for seed oil synthesis has great significance for soybean improvement. In this study, we found that oil accumulation rates and gene expression levels changed dynamically during soybean seed development. The expression levels of genes in metabolic pathways such as carbon fixation, photosynthesis, glycolysis, and fatty acid biosynthesis were significantly up-regulated during the rapid accumulation of oil in developing soybean seeds. Through weighted correlation network analysis, we identified six co-expression modules associated with soybean seed oil content and the pink module was the most positively correlated (*r* = 0.83, *p* = 7 × 10^−4^) network. Through the integration of differential expression and co-expression analysis, we predicted 124 candidate genes potentially affecting soybean seed oil content, including seven genes in lipid metabolism pathway, two genes involved in glycolysis, one gene in sucrose metabolism, and 12 genes belonged to transcription factors as well as other categories. Among these, three genes (*GmABI3b, GmNFYA and GmFAD2-1B*) have been shown to control oil and fatty acid content in soybean seeds, and other newly identified candidate genes would broaden our knowledge to understand the molecular basis for oil accumulation in soybean seeds.

## 1. Introduction

Soybean (*Glycine max* L. Merr.) has been recognized as one of the most vital economic, as well as potential bioenergy crops. It provides a significant amount of protein for both humans and animals, and soybean seed oil is a significant source for vegetable oil and industrial materials. World soybean production in 2017 was over 340 million metric tons, and soybean oil accounts for 29% of world vegetable oil consumption (www.soystats.com). Due to the population growth, the demand for food oil continues to grow. Increasing soybean oil content has become an important topic in scientific research and a key target trait for soybean breeders.

In plants, the oil formation process is composed of four steps: fatty acid de novo synthesis, acyl elongation and editing, triacylglycerol (TAG) assembly and oil drop formation [1], and each step involves many genes. It has been suggested that the number of genes involved in lipid signaling and membrane lipid synthesis were two to three-fold higher, and 63% more genes involved in the plastid de novo fatty acid synthesis in soybean than Arabidopsis. Many single-member enzymes in Arabidopsis have multiple homologs in soybean [2]. For instance, there is only one gene (*At2g30200*) encoding malonyl-CoA: ACP malonyltransferase (MCMT) but two genes (*Glyma.11G164500* and *Glyma.18G057700*) encoding MCMT in soybean. In total, there are 1127 putative acyl lipid related genes in the soybean genome [2]. Even though the in-depth study of fatty acid metabolism in model plant Arabidopsis lays a foundation for the study of lipid synthesis in other plants [3], the fatty acid metabolic pathways in soybean remain to be elucidated.

The whole genome expression profile plays an important role in exploring candidate genes and investigating complex metabolisms [4]. Some lipid-related candidate genes have been identified by the transcriptome analysis of *Brassica napus* pods [5]. However, traditional differential gene expression analysis only compares the transcriptional changes between two samples each time, while the relationships between genes are not investigated. Genes with similar expression patterns may be co-regulated, functionally related or in the same pathway [6,7]. Gene co-expression network (GCN) analysis can simultaneously analyze the gene expression data of all samples to effectively identify functionally co-expressed genes. It is especially suitable for the study of complex large-scale gene expression data, such as different developmental stages of the same tissue [8], different organs or tissues [9,10], responses at different time points after abiotic stress [11] and pathogen infection [12]. GCN analysis includes three steps [13]: First, the relationships between genes are determined by various measurements such as Pearson’s correlation. Second, the associations of genes are used to construct a network, where the genes are connected with each other with each node represents a gene and each edge indicates the strength of the relationship. Third, the co-expressed genes are identified using the available clustering methods such as *k*-means or hierarchal clustering.

With the development of GCN analysis, it is suggested setting a threshold for Pearson’s correlation coefficient to determine the existence of a network [14]. To pick the appropriate threshold, Zhang and Horvath proposed a new framework for ‘soft’ thresholding that weighs each connection, which is called the weighted correlation network [6], and constructs the co-expression network more consistently with the scale-free network distribution and biological significance. The weighted correlation network analysis (WGCNA) [15] is based on the new framework and follows the steps of GCN analysis as described above. WGCNA adopts topological overlap measure (TOM) to calculate the degree of association between genes, and identifies clusters (modules) of highly correlated genes (co-expressed gene network). The central nodes (the ones with most connections to other nodes) in the network are referred to as hub genes. The gene expression profile in a module is represented by the module eigengene (ME). The module membership (MM) quantifies how close a gene is to the module and the hub genes tend to have high MM values to their respective modules. After GCN is constructed, the major goal is to find the biologically significant modules and genes. The correlation between the individual gene and a biological trait is defined as the gene significance (GS), and the average GS across the genes in a module is module significance [15]. Therefore, the higher values of GS and module significance, the more biologically significant is a gene and module. WGCNA has been used to uncover a network module containing 34 genes, which was highly correlated (*r* = 0.95, *p* = 9.0 × 10^−13^) with apple anthocyanin contents [8]. Another study identified eight key genes with direct impact on biosynthesis and accumulation of three flavonoid compounds during the flower development of *Camellia sinensis* using WGCNA [16].

Several groups have studied the transcriptome profiles in developing soybean seeds to analyze the patterns of differently expressed genes (DEGs) during soybean seed development [17,18], identifying the transcript sequence polymorphisms (including SNPs, small Indels and large deletions) among soybean varieties differing in oil content and composition [19,20], screening candidate genes related to oil synthesis based on gene expression patterns among different tissues or in seeds of developmental stages [21,22], and understanding the genetic basis underlying soybean domestication [23,24]. These studies have contributed greatly to dissecting the oil synthesis process in soybean seeds. However, the dynamic changes in transcriptome related to oil accumulation during soybean seed development are still not well understood, and the correlation between transcriptomic changes with seed oil content needs to be explored.

In this study, the total oil content in developing soybean seeds at different developmental stages were determined to characterize the oil accumulation pattern. Then the ovules at 0 days after flowering (0 DAF, the first day of flowering) and developing seeds at 10, 20, 30, and 40 DAF were subjected to RNA sequencing (RNA-Seq) aiming to identify the transcriptomic changes and DEGs during seed development. Further, WGCNA was conducted to investigate the network and candidate genes highly associated with soybean seed oil synthesis. This work will contribute to a better understanding of the molecular basis of oil synthesis during soybean seed development.

## 2. Results

### 2.1. Dynamic Changes of Oil Content in Developing Soybean Seeds

**Figure 1:**
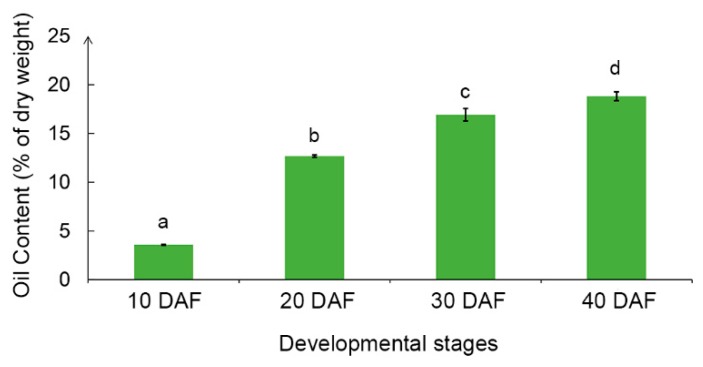
Total oil content in developing soybean seeds of NN1138-2 at 10, 20, 30, and 40 days after flowering (DAF). Different letters above the bars represent significant differences at 0.05 level by Fisher’s least significant difference (LSD) test. Error bars represent the standard deviation (SD) of means across three replications.

The total oil content increased along with the soybean seed development (Figure 1). At 10 DAF, there was a very low level of total oil content, which was less than 5% of dry weight. But when reaching 20 DAF, the total oil content in seeds dramatically increased to 12.7%. At 30 DAF and 40 DAF, soybean seed accumulated 16.9% and 18.8% oil, respectively (Figure 1). From 10 DAF to 20 DAF was the critical period for rapid accumulation of oil with the oil content increasing by 9.11% within 10 days.

### 2.2. DEGs Between Adjacent Developmental Stages and Validation of RNA-Seq by Quantitative RT-PCR

**Figure 2:**
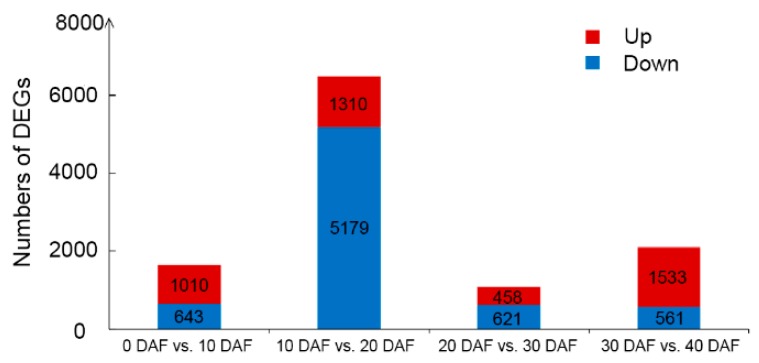
Numbers of differently expressed genes (DEGs) in comparison with the soybean developing seeds between adjacent developmental stages. DEGs were determined using the thresholds of *FDR* ≤ 0.05 and log2|Fold change| ≥ 1. The log2|Fold change| value of the pairwise comparison “a DAF vs. b DAF” was calculated by the formula: log2|Fold change| = log2(FPKM__b DAF_)-log2(FPKM__a DAF_).

To investigate the dynamic changes in transcriptome during soybean seed development, we analyzed the developing seeds at 10, 20, 30 and 40 DAF as well as the ovules at 0 DAF. We compared the numbers of DEGs (FDR ≤ 0.05 and log2|Fold change| ≥ 1) in comparison with the samples at adjacent developmental stages including a total of four pairwise comparisons (Figure 2). The total number of DEGs ranged from 1079 (20 DAF vs. 30 DAF) to 6489 (10 DAF vs. 20 DAF). The comparison of 10 DAF vs. 20 DAF had the largest number of DEGs, which reflects the phenotypic changes in the total oil content from 10 DAF to 20 DAF (Figure 1).

To verify the results of RNA-Seq, we randomly selected eight genes from those with FPKM > 0 in at least two stages for qRT-PCR. The qRT-PCR results of these genes showed consistency with the RNA-Seq data, indicating a good reliability of our RNA-Seq results (Figure S1).

### 2.3. Dynamic Changes in Expression Levels of Genes in the Fatty Acid Metabolic Pathways in Developing Soybean Seeds

**Figure 3:**
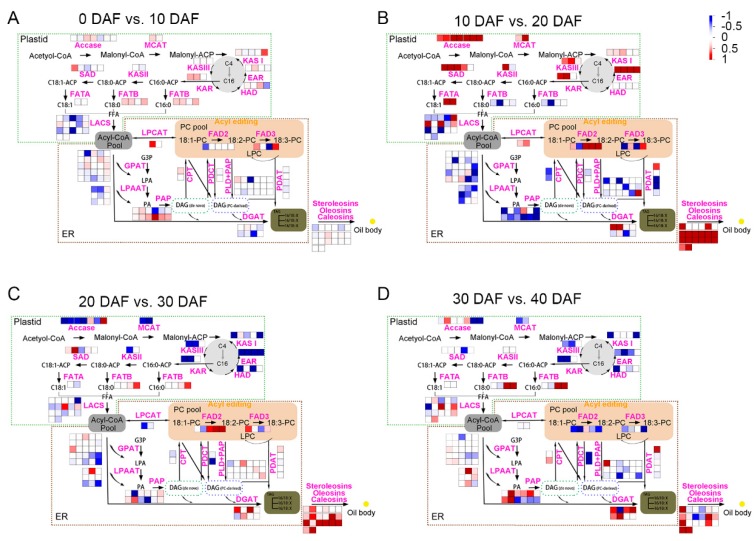
Mapman visualization of differentially expressed genes in the lipid metabolic pathways between the adjacent developmental stages in soybean seeds. (**A**) 0 DAF vs. 10 DAF. (**B**) 10 DAF vs. 20 DAF. (**C**) 20 DAF vs. 30 DAF. (**D**) 30 DAF vs. 40 DAF. Every square represents a gene. The color represents the log2|Fold change| of the gene with down-regulation in blue while up-regulation in red. The dotted boxes represent plastid and endoplasmic reticulum (ER), respectively. ACCase: acetyl CoA carboxylase. CPT: cholinephosphotransferase. DGAT: diacylglycerol: acyltransferase. EAR: enoyl-ACP reductase. FAD2: Fatty desaturase 2. FAD3: Fatty desaturase 3. FATA: fatty acyl-ACP thioesterase A. FATB: fatty acyl-ACP thioesterase B. GPAT: glycerol-3-phosphate acyltransferase. HAD: hydroxyacyl-ACP dehydrase. KAR: ketoacyl-ACP reductase. KAS I: ketoacyl-ACP synthase I. KAS II: ketoacyl-ACP synthase II. KAS III: ketoacyl-ACP synthase III. LACS: long chain acyl-CoA synthetase. LPAAT: lysophosphatidic acid-acyltransferase. LPCAT: lysophosphatidylcholine acyltransferase. MCMT: malonyl-CoA: ACP malonyltransferase. PAP: phosphatidic acid phosphatase. PDAT: phospholipid: diacylglycerol acyltransferase. PDCT: phosphatidylcholine: diacylglycerol choline phosphotransferase. PLD: phospholipase D. SAD: stearoyl-ACP desaturase.

Pathway analysis is useful to help us understand the dynamic changes in oil synthesis-related DEGs during oil accumulation process in soybean seeds. Formation of oil in plant seeds has gone through the following process: de novo synthesis and elongation of fatty acids in plastid, desaturation of fatty acids and assembly of TAG in endoplasmic reticulum (ER), and finally formation of oil bodies. Therefore, we identified the genes encoding the known enzymes in the fatty acid metabolism, and then used MapMan to visualize the expression level changes of these genes in soybean seeds at adjacent developmental stages (Figure 3). Consistent with Figure 2, there were also fewer DEGs in lipid metabolic pathways between 0 DAF vs. 10 DAF (Figure 3A). The levels of most genes in the de novo synthesis and elongation of fatty acids in plastid were higher at 20 DAF than 10 DAF (Figure 3B), including Acetyl-CoA carboxylase (ACCase) and Malonyl-CoA: ACP malonyltransferase (MCMT) which were responsible for catalyzing the synthesis of fatty acid precursors, the ketoacyl-ACP synthase family I, III (KAS I and KAS III), ketoacyl-ACP reductase (KAR), hydroxyacyl-ACP dehydrase (HAD) and enoyl-ACP reductase (EAR) that were responsible for elongation of the precursors carbon chains from C4 to C16. All of the *oleosin* genes responsible for oil body formation also showed significant up-regulation at 20 DAF (Figure 3B). Although soybean seed oil content continued to increase at 30 DAF and 40 DAF, the increasing rate was slower than that at 20 DAF (Figure 1). More genes in the fatty acid synthesis pathway showed to be down-regulated at 30 DAF compared with 20 DAF (Figure 3C), and at 40 DAF compared with 30 DAF (Figure 3D), reflecting the expression levels of these fatty acid synthesis related genes gradually decreasing after 20 DAF, which coincides with the slower oil accumulation rate after 20 DAF.

### 2.4. Functional Analysis of DEGs Comparing 20 DAF with 10 DAF

**Figure 4:**
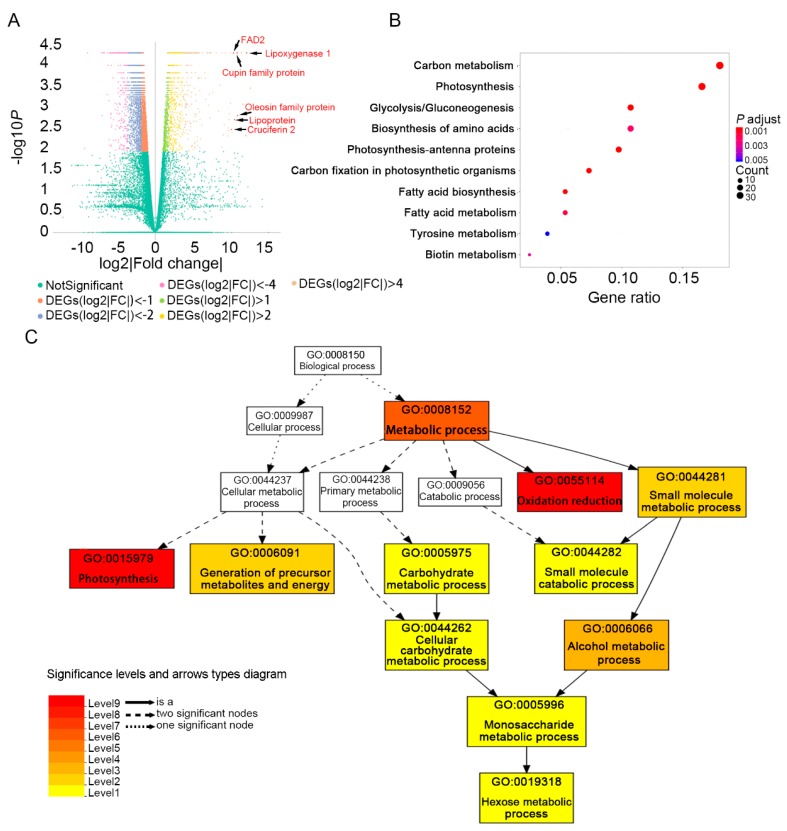
Functional analysis of DEGs comparing 20 DAF with 10 DAF in developing soybean seeds. (**A**) Volcano plot of differentially expressed genes. The x-axis shows log2|Fold change| in expression and the y-axis represents the minus log10 (*p* value) of a gene being differentially expressed. log2|Fold change| = log2 (FPKM__20 DAF_)- log2(FPKM__10 DAF_). Genes related to lipid metabolism or lipid storage in the top 10 up-regulated DEGs at 20 DAF vs. 10 DAF are marked by red points and black arrows. (**B**) Top 10 enriched KEGG pathways of up-regulated genes comparing 20 DAF with 10 DAF (*p* < 0.01). The colors of dots represent the *p* values relative to the other displayed pathways and the dot size represents the number of genes in the pathway. The x-axis shows gene ratio and y-axis shows the pathway. Gene ratio is the ratio of up-regulated gene number (comparing 20 DAF with 10 DAF) to the soybean genome-wide number in a certain pathway. (**C**) Gene ontologies (GO) enrichment analysis (in biological process) of up-regulated genes comparing 20 DAF with 10 DAF (*FDR* < 0.01). The numbers and names of GO terms are shown in boxes. Box colors indicate levels of statistical significance: Level 1 *p* = 0.05, Level 2 *p* = 5 × 10^−3^, Level 3 *p* = 5 × 10^−4^, Level 4 *p* = 5 × 10^−5^, Level 5 *p* = 5 × 10^−6^, Level 6 *p* = 5 × 10^−7^, Level 7 *p* = 5 × 10^−8^, Level 8 *p* = 5 × 10^−9^, Level 9 *p* = 5 × 10^−10^. Solid, dashed, and dotted lines represent two, one and zero enriched terms at both ends connected by the line, respectively.

As the seed oil content showed a significant increase from 10 DAF to 20 DAF (Figure 1), and the number of DEGs between these two stages was larger than other groups in comparison of adjacent stages, we further analyzed the DEGs of “10 DAF vs. 20 DAF”. In total, there were 6489 DEGs between 10 DAF and 20 DAF. Among them, 1310 genes were up-regulated and 5179 genes were down-regulated (Figure 2). The top 10 DEGs (*FDR* ≤ 0.05) with the highest value of log2|Fold change| comparing 20 DAF with 10 DAF are shown in Table S1. Strikingly, genes encoding proteins involving in lipid storage or lipid metabolism such as lipoxygenase, cupin family protein, oleosin family protein, lipoprotein and FAD2 were up-regulated at 20 DAF (Figure 4A, Table S1). Oleosin and FAD2 were recognized as important proteins involved in the fatty acid desaturation and oil droplets formation [25,26]. Although lipoxygenase is not directly involved in the synthesis of fatty acids, it can oxidize polyunsaturated fatty acids [27].

Kyoto Encyclopedia of Genes and Genomes (KEGG) pathway enrichment analysis of DEGs comparing 20 DAF with 10 DAF (Figure 4B, Table S2) showed that the up-regulated genes were enriched in 14 pathways (Table S2) and the top 10 enriched pathways (Figure 4B) include “carbon metabolism (gmx01200)”, “photosynthesis (gmx00195)”, “glycolysis (gmx00010)”, “photosynthesis-antenna proteins (gmx00196)”, “carbon fixation in photosynthetic organisms (gmx00710)”, “fatty acid biosynthesis (gmx00061)” and “fatty acid metabolism (gmx01212)”, which were not enriched in the down-regulated DEGs (Table S2). The down-regulated DEGs were mainly enriched in pathways (Table S2) related to DNA replication and repair such as “purine metabolism (gmx00230)”, “DNA replication (gmx03030)”, and “nucleotide excision repair (gmx03420)”.

The gene ontology (GO) classification of these DEGs was compared against that of all genes in soybean genome. The up-regulated DEGs were enriched in 11 GO terms of biology process as shown in Figure 4C and Table S2. The most significant enrichment GO terms were “photosynthesis (GO: 0015979)” and “oxidation reduction (GO: 0055114)” including 34 and 133 genes, respectively. The other enriched GO terms in the up-regulated genes included “monosaccharide metabolic process (GO:0005996)”, “carbohydrate metabolic process (GO:0005975)”, “hexose metabolic process (GO:0019318)” and “cellular carbohydrate metabolic process (GO:0044262)”, which were related to carbon metabolism. Similar to the KEGG enrichment result, the down-regulated genes were not enriched in terms related to fatty acid synthesis (Table S2).

### 2.5. Co-Expression Gene Networks and Their Correlations with Soybean Seed Oil Content

**Figure 5:**
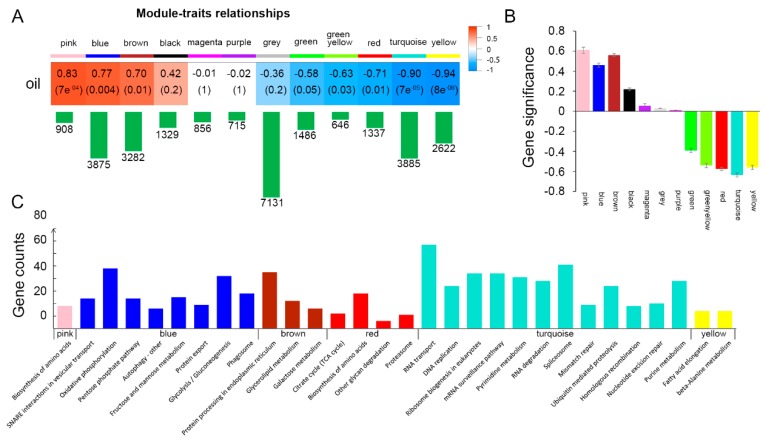
Correlation between gene co-expression network and oil content in developing soybean seeds. (**A**) The correlations between gene co-expression modules and oil content. The first colored row represents the modules detected by Weighted Correlation Network Analysis (WGCNA), and the colored cells below represent their correlations with oil content. The corresponding correlation coefficient (top) and *p* value (bottom) are displayed in each cell. The cells are color-coded by the correlation coefficient (*r*) as shown by the color key on the right. Red represents positive correlation while blue represents negative correlation. The green bar below each module represents the number of genes in each module. (**B**) Bar plot represents the average gene significance for each detected module. (**C**) Enriched pathways in genes of pink, blue, brown, red, turquoise and yellow modules, respectively. The x-axis shows the names of the pathways, and the y-axis displays the gene numbers in each pathway.

Co-expression gene networks were analyzed across all samples in this study. A total of 12 modules (in different colors) were identified, containing ~646-7131 genes per module (Figure 5A). Expression patterns of the 12 modules were shown in Figure S2. Among them, the pink, blue and brown modules containing 908, 3875 and 3282 genes, respectively, showed significant (*p* ≤ 0.01) positive correlation with soybean seed oil content. The red, turquoise and yellow modules with 1337, 3885 and 2622 genes respectively showed significant (*p* ≤ 0.01) negative correlation with seed oil content. The gene significance (GS) was defined as the correlation between the gene expression and phenotype [28]. Therefore, the higher GS absolute value represents the higher correlation between the gene expression pattern and the phenotype. Modules that are significantly associated with oil content have higher average GS absolute values than others (Figure 5B).

As the pink, blue, brown, yellow, turquoise and red modules were significantly correlated with soybean seed oil content, pathway enrichment analysis was performed for these six groups of genes. It was found that genes in different modules were enriched in distinct pathways (Figure 5C). Pink module showed a high correlation (*r* = 0.83, *p* = 7 × 10^−4^) with oil content, which was enriched in “biosynthesis of amino acids (gmx01230)”. Many of these genes also belonged to the “glycolysis” pathway, such as *fructose*-*bisphosphate aldolase* (*Glyma.02G222400*) and *NADH glutamate synthase* (*Glyma.19G065600*). Amino acids serve as important energy sources through their catabolism via the tricarboxylic acid cycle (TCA) cycle, and researchers found that the contributions of amino acid catabolism to the energy requirements of developing seeds appears even more critical than in vegetative tissues due to the limits of oxygen diffusion [29].

The blue module (*r* = 0.77, *p* = 0.004) was significantly enriched in eight pathways. Among them, the “pentose phosphate pathway (gmx00030)”, “glycolysis/gluconeogenesis (gmx00010)” and “fructose and mannose metabolism (gmx00051)” belong to carbohydrate metabolism. It is known that glucose is converted to pyruvate via glycolysis, and then generate acetyl-CoA as the precursor of fatty acids. Besides that, ATP produced during glycolysis could also be utilized for fatty acid synthesis. The pentose phosphate is a parallel pathway to glycolysis and could provide NADPH^+^ and H^+^. In most creatures, sugar and fat could be converted to each other, and fructose and glucose are required in TAG accumulation. Oxidative phosphorylation (gmx00190) could also generate ATP. It is likely that the genes in the blue module are responsible for providing the carbon source and energy for the synthesis of fatty acids (Figure 5C).

The genes in the brown module (*r* = 0.70, *p* = 0.01) are enriched in glycerolipid metabolism pathway (gmx00561) by which the glycerolipids such as diacylglycerol (DAG) and triacylglycerols (TAGs) are generated. As a metabolic pathway directly related to TAG synthesis, homologous of genes in this module are directly related to fatty acid synthesis such as phospholipid:diacylglycerol acyltransferase (PDAT) [30] and diacylglycerol kinase (DGK) [31] (Figure 5C).

The turquoise module negatively correlated (*r* = −0.9, *p* = 7 × 10^−5^) with seed oil content, which containing genes enriched in 12 pathways that are related to DNA/RNA processing and proteolysis. These are similar with the GO enrichment of DEGs comparing 20 DAF with 10 DAF, that the enriched terms of down-regulated genes are mainly related to DNA replication and repair (Figure 5C). These results suggest that DNA replication may slow down when oil is accumulated rapidly.

### 2.6. Identification of Hub Genes in the Co-Expression Modules Related to Soybean Seed Oil Content

**Figure 6:**
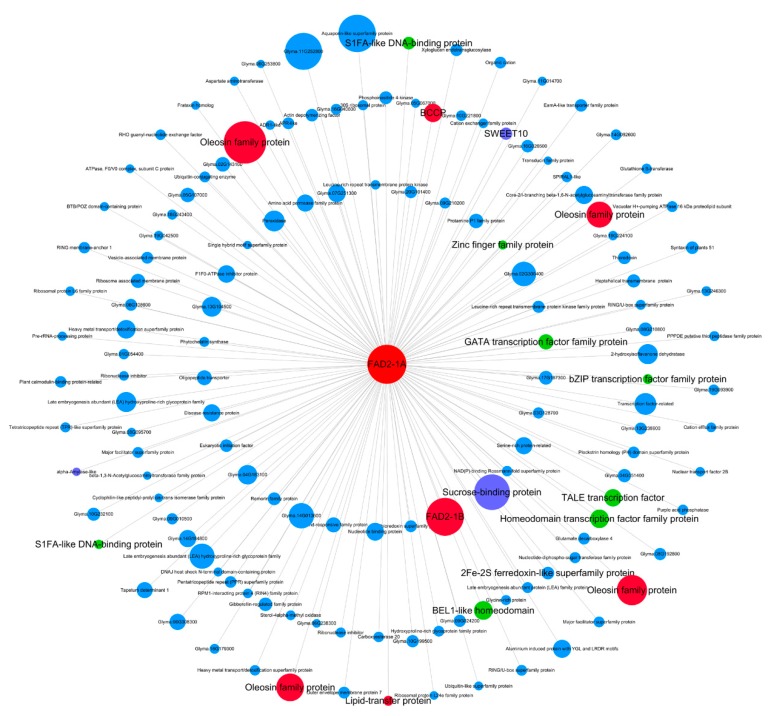
Co-expression network for *FAD2-1A* (*Glyma.10G27800*) in the pink module. Only the genes (nodes) with values of gene significance (GS) to oil content, the module membership (MM) to pink module and the gene expression pattern correlation (*r*) with *FAD2-1A* are all greater than 0.8 are shown. The node size represents the value of log2|Fold change| comparing 20 DAF with 10 DAF. The highlighted red nodes represent lipid metabolism related genes, the green nodes represent transcription factors, the purple nodes represent sucrose and starch metabolism related genes. The innermost genes represent correlation coefficient with *FAD2-1A* expression greater than 0.95, the middle layer genes represent correlation with *FAD2-1A* expression between 0.90 and 0.95, and the outer layer genes represents correlation with *FAD2-1A* between 0.8 and 0.9.

Hub genes are often considered as important factors in gene co-expression networks. Here we identified the top 10 hub genes in each of the pink, blue, brown, yellow, turquoise and red modules (Table S3) according to module membership (MM) value, which is also known as *kME* (eigengene connectivity). Among these hub genes, *FAD2-1A* (*Glyma.10G278000*) responsible for converting C18:1 to C18:2 [32] is in the pink module with the highest MM value (Table S3). The visual co-expressed network of *FAD2-1A* (*Glyma.10G278000*) in the pink module was constructed (Figure 6). The *FAD2-1A* co-expressed genes include genes encoding four oleosins (*Glyma.05G013800*, *Glyma.06G078700*, *Glyma.06G209900* and *Glyma.17G122000*), the FAD2-1B (*Glyma.20G111000*), a biotin carboxyl carrier protein (*BCCP*, *Glyma.19G028800*) and a lipid transfer protein (*Glyma.16G100100*), which are related to lipid metabolism. This co-expression network also contains genes encoding eight transcription factors (*Glyma.01G179900*, *Glyma.03G179000*, *Glyma.05G209600*, *Glyma.11G062300*, *Glyma.17G132600*, *Glyma.19G179700*, *Glyma.U018600*, and *Glyma.06G047000*), a sweet sucrose efflux transporter family protein (*Glyma.06G167000*), a sucrose binding protein (*Glyma.02G145700*) and an alpha-amylase (*Glyma.14G222600*), which are related to sucrose and starch metabolism.

*11-β-hydroxysteroid dehydrogenase-like* (*HSD*, *Glyma.11G015100*) in the blue module had the highest up-regulation ratio comparing 20 DAF with 10 DAF among all hub genes (Table S3). It was also found that *HSDs* are minor components of oil bodies in oilseeds [33]. Overexpressing *AtHSD* showed increased seed yield as well as a reduced seed dormancy [34].

The turquoise module was negatively correlated with oil content in developing soybean seeds, and the pathway enrichment analysis suggested this module might be associated with cell division process (Figure 5). In this module, we found a cyclin-dependent kinase gene (*Glyma.17G262300*) as the hub gene (Table S3). This protein has been confirmed to regulate cell division cycle and have also been implicated in the control of gene transcription and other processes [35].

### 2.7. Identification of Candidate Genes Related to Soybean Seed Oil Synthesis

The WGCNA analysis found that three modules, pink (*r* = 0.83, *p* = 7 × 10^−4^), blue (*r* = 0.77, *p* = 0.004) and brown (*r* = 0.7, *p* = 0.01), had significant positive correlations with oil content, containing 908, 3875 and 3282 genes, respectively. We further identified 124 key candidate genes related to soybean seed oil synthesis from these three modules by screening DEGs with GS values greater than 0.8 and log2|Fold change| greater than 2 (*FDR* < 0.05) comparing 20 DAF with 10 DAF (Table S4, Figure S3). Among these candidate genes, 12 of them (*Glyma.06G047000*, *Glyma.17G132600*, *Glyma.02G058800*, *Glyma.02G303800*, *Glyma.06G290100*, *Glyma.07G038400*, *Glyma.08G357600*, *Glyma.12G236800*, *Glyma.16G007400*, *Glyma.05G056000*, *Glyma.07G060400* and *Glyma.10G071700*) belong to transcription factors, seven of them (*Glyma.13G010100*, *Glyma.19G028800*, *Glyma.13G057400*, *Glyma.18G202800*, *Glyma.07G268500*, *Glyma.20G111000* and *Glyma.16G058100*) involve in fatty acid biosynthesis and glycerolipid metabolism, two of them (*Glyma.13G035200* and *Glyma.19G000700*) involve in glycolysis, and *Glyma.19G212800* is involved in starch and sucrose metabolism. Among these 124 key candidate DEGs (Table S4), a total of 41 genes were more highly expressed in soybean seed than other tissues (leaf, flower, pod, root and nodule) and five of them (*Glyma.01G227900*, *Glyma.03G229700*, *Glyma.08G357600*, *Glyma.10G064300* and *Glyma.19G002400*) were seed-specific expressed genes (Table S4). *Glyma.08G357600* encodes an *abscisic acid-insensitive 3* (*ABI3*) transcription factor, which has been designated as *GmABI3b* and found to activate the expression of *GmWRI1a* by direct binding to the RY motif of *GmWRI1a* promoter to regulate soybean seed oil content [36], and its homologue in *Arabidopsis thaliana* is also involved in seed oil biosynthesis [37].

## 3. Discussion

Seed oil content is an important agronomic trait of soybean. However, the molecular basis of oil accumulation and genes related to oil synthesis in soybean seeds has not been well understood. The correlation between changes in transcriptome and oil content in soybean seeds has not been explored. Here in this study, we associated the dynamic changes in transcriptomes with phenotypic changes (oil content) during soybean seed development, and proposed the potential important pathways and candidate genes affecting the seed oil content in soybean.

### 3.1. Pathways Associated with Soybean Seed Oil Synthesis

As the soybean seed oil content showed a significant increase from 10 DAF to 20 DAF (Figure 1), we performed KEGG pathway enrichment analysis of DEGs comparing 20 DAF with 10 DAF and found that carbon metabolism, glycolysis and photosynthesis are the top three significantly enriched pathways (Figure 4B). The blue and brown modules identified by WGCNA that showed positive correlations with oil content were also enriched in carbohydrate metabolism related and glycerolipid metabolism pathways. Both carbohydrate and glycolysis metabolism were confirmed to provide carbon sources for fatty acid synthesis [1,38]. Fatty acids are synthesized in seed plastids using the sucrose from photosynthesis. Although seed is not the main tissue for photosynthesis, the young green immature seeds also have photosynthesis ability [39]. We propose that in the process of soybean seed oil synthesis and accumulation, a large amount of carbon source is needed. The fixated carbon in leaves needs to be transported to seeds through the phloem, while the sucrose fixed by the immature seeds can directly supply the carbon sources for fatty acid synthesis in seeds, which may improve the efficiency of converting carbon into fatty acids. It has been found that embryonic photosynthesis provides energy and oxygen in developing seeds, which could increase the biosynthetic fluxes to lipids [40].

In addition, the DEGs of 20 DAF vs. 10 DAF were also enriched in fatty acid synthesis and metabolism pathways (Figure 4B). Fatty acid synthesis and TAG assembly are the pathways directly related to oil accumulation. The expression levels of most genes in the fatty acid synthesis pathway and a few genes involved in TAG assembly were significantly up-regulated comparing 20 DAF with 10 DAF (Figure 3B). In the fatty acid de novo synthesis pathway, ACCase controls the first committed step. Overexpression of *ACCase* gene would increase oil content in plants [41,42]. Also, the modulation of *KAS II* levels would change seed fatty acid composition in Arabidopsis [43]. In our results, the *ACCase* genes (*Glyma.08G027600* and *Glyma.05G221100* encoding *biotin carboxylase subunit* and *Glyma.13G057400*, *Glyma.18G265300* and *Glyma.19G028800* encoding *biotin carboxyl carrier protein subunit*) were all up-regulated at 20 DAF vs. 10 DAF (Figure 3B). And the expression level of *Glyma.17G047000* encoding *KAS II* increased at 20 DAF vs. 10 DAF (Figure 3B). Among the eight *GmDGATs* in the TAG assemble pathway, expression of *Glyma.13G106100* and *Glyma.09G065300* significantly increased at 20 DAF vs. 10 DAF (Figure 3B). A previous report had found that *GmDGAT1A* (*Glyma.13G106100*) was highly expressed in seeds and overexpression of it in Arabidopsis seeds would enhance the TAG production [44].

The down-regulated DEGs at 20 DAF vs. 10 DAF were enriched in the pathways mainly related to DNA replication and repair (Table S2). The turquoise module having a negative correlation with oil content (*r* = −0.9, *p* = 7 × 10^−5^) was also enriched in DNA replication and repair related pathways. The *cyclin-dependent kinase* (*Glyma.17G262300*) was the hub gene identified in the turquoise module, which has the annotations of regulating the cell cycle, transcription, mRNA processing, and differentiation of cells. We suspect that the rate of oil accumulation may be negatively correlated with cell differentiation.

### 3.2. Screening Soybean Seed Oil Related Candidate Genes Based on WGCNA Analysis

With the rapid development of high-throughput sequencing technology, researchers usually use multi-samples for RNA-Seq to study complex metabolic mechanisms. However, the traditional pairwise comparisons cannot effectively reflect the overall dynamic characteristics of all samples. Previous studies have shown that by using WGCNA, massive transcriptome data could be effectively utilized to classify genome-wide genes into gene co-expression modules, then to further study the relevance between co-expression modules and target traits. This method is especially suitable for the study of multiple samples at different developmental stages or different treatments [10,45]. The hub genes that highly interconnected with nodes in a module, have been generally considered functionally significant [46]. In our study, we identified 6 modules (Figure 5A) that were highly associated with oil content in developing soybean seeds, and the pink module has the highest positive correlation with seed oil content (*r* = 0.83, *p* = 7 × 10^−4^). The top hub gene in the pink module is the *FAD2-1A* that catalyzes the synthesis of linoleic acid, which is a major component of fatty acids in soybean seeds.

In addition, GS values based on the correlation of a gene expression profile with sample trait calculated by WGCNA were efficiently used to identify key candidate genes. GS of a node can defined as the correlation between the node and the phenotypic trait [28]. Combining the GS values greater than 0.8 in the three modules (pink, blue and brown) showing significant positive correlation with oil content, and log2|Fold change| greater than 2 (*FDR* < 0.05) comparing 20 DAF with 10 DAF, totally we identified 124 candidate genes associated with soybean seed oil content, of which two genes, *GmNFYA* (*Glyma.02G303800*) [23] and *GmFAD2-1B* (*Glyma.20G111000*) [32] have been confirmed to control soybean seed fatty acid content in previous studies, and the other newly identified candidate genes would broaden our knowledge to understand the molecular basis for oil accumulation in soybean seeds.

In addition to oil content, many other traits such as the content of protein, isoflavones, starch, and tocopherol in seeds also change during seed development. Previous studies showed that soybean seed oil content has a strong negative correlation (*r* = −0.75; *p* < 0.0001) with seed protein content [47], negative correlation (*r* = −0.427; *p* < 0.01) with isoflavones content [48], positive correlation (*r* = 0.1295; *p* < 0.05) with starch content [49], negative correlation (*r* = −0.371; *p* < 0.01) with alpha-tocopherol and negative correlation (*r* = −0.391; *p* < 0.01) with beta-tocopherol [50]. In addition, some pleiotropic loci have also been identified [47,51,52]. The correlation between gene expression and other traits as well as the correlation between different traits during soybean seed development would help us to identify the important pleiotropic genes, which should be investigated in future research. Also, transcriptomic comparisons between different soybean lines with low and high oil content would provide additional information to further select key genes controlling seed oil content, which is also worth to pursue in the next study.

## 4. Materials and Methods

### 4.1. Plant Materials and Sample Collection

The seeds of soybean variety ‘nannong1138-2′ (NN1138-2) were planted in the experimental station of Nanjing Agricultural University. The days after flowering (DAF) of pods were marked with tags to track the development of seeds inside the pods.

Samples at the five development stages, including the ovules at 0 DAF, and developing seeds at 10, 20, 30, and 40 DAF with three biological replications were collected. All samples for fatty acid determination were frozen in the liquid nitrogen and then stored at −80 °C, while the ones for RNA-Seq were immersed in the RNA-later reagent (Cat. no. AM7020, Invitrogen™, Waltham, Massachusetts, USA) and kept at 4 °C overnight before moved to −80 °C refrigerator for storage.

### 4.2. Quantitation of Seed Oil Content

The oil content was determined by the Gas Chromatograph (GC, Thermo Scientific™, Waltham, Massachusetts, USA) according to the previously published method [22]. The CP-Sil 88 (Agilent Technologies, Santa Clara, California, USA) was used as the GC column. Nitrogen gas of 35 Kpa was used as carrier gas, air pressure was set to 350 Kpa, and hydrogen pressure was set to 30 Kpa. The GC temperature programming was set as the following: the injection port temperature was 200 °C, detector temperature was 270 °C. The initial temperature was set to 40 °C for a duration of 1 min and then raised to 350 °C at a temperature increase rate of 10 °C/min. The split ratio was 1:15.

The samples were ground with liquid nitrogen in the mortar and then dried in the vacuum freezing dryer for 24 h until the weight didn’t change anymore. Next, 100 mg powder sample was into a 2 mL centrifuge tube with 1% heptadecanoic acid as internal standard in it. The sample was then immersed in the 1 mL methylation agent (2.5%, *v*/*v*, H_2_SO_4_ in CH_3_OH) and incubated at 85 °C for 1 h in the water bath for methyl esterification. After that, the extraction was centrifuged, and then the supernatant was retained, and then mixed with 600 µL 0.9% (*w*/*v*) NaCl and 350 µL n-hexane later. The mixture was centrifuged for 10 min at 4000 rpm and the organic phase (supernatant liquid) was air-dried. Lastly, we dissolve the dried methyl esterification samples in 500 µL ethyl acetate and subjected to GC.

### 4.3. Library Construction and RNA Sequence

Total RNA was isolated using the RNA Isolation Kit (Cat. no. AM1561, Invitrogen™, Waltham, Massachusetts, USA) according to the manufacturer’s instructions. The minimum total amount of RNA used for RNA-Seq was 3 μg and the concentration was more than 50 ng/μL for all samples. RNA quality was checked by the Agilent 2100 Bioanalyzer (Agilent Technologies, Santa Clara, California, USA) to meet the criteria of OD_260/280_ ≧ 1.8, 28S/18S ≧ 1, and RNA Integrity Number (RIN) ≧ 7. RNA-Seq library was constructed according to the instructions of NEBNext^®^ Ultra™ RNA Library Prep Kit for Illumina (Cat. no. E7530L, NEB, Ipswich, Massachusetts, USA). RNA paired-end (PE) sequencing was performed on the Illumina HiSeq2500 sequencer (Illumina, San Diego, California, USA) at the National Key Laboratory of Crop Genetics and Germplasm Enhancement, Nanjing Agricultural University, and the sequencing length was 101 bp. All of the RNA-seq raw data have been submitted to the NCBI BioProject with the SRA accession number PRJNA539842.

### 4.4. Raw Data Filtering

Cutadapt (Version 1.16) software [53] was used to remove adapters and overrepresented sequences. Then the Trimmomatic (Version 0.38) [54] was used to filter the low-quality bases or N bases, the average read quality score threshold was set to 20 using a four-base sliding window, the minimum read length is set to 25 [55]. The qualities of raw data and clean data were controlled by FastQC (http://www.bioinformatics.babraham.ac.uk/projects/fastqc/).

### 4.5. Transcriptome Analysis and Data Normalization

The sequence and corresponding annotation of reference soybean genome Williams 82 v2.0 were downloaded from the Phytozome v12.0 database (https://phytozome.jgi.doe.gov) [56]. The soybean reference genome was indexed using bowtie-build (version 1.2.2) [57]. Then clean reads of 15 samples were all aligned to the reference genome using Tophat (Version 2.0.13) [58] allowing no more than two nucleotide mismatches. The abundance of transcripts was estimated using Cufflinks (v2.2.1) [58]: cufflinks was used to assemble new transcripts and the transcript abundance was indicated by the fragments per kilobase per million (FPKM), then cuffmerge was used to merge all transcripts removing redundancy. Finally, cuffdiff was used to calculate the fold change and *FDR* values of DEGs. Volcano maps for DEGs were drawn by ggplot (version 3.0.0) and ploty (version 4.8.0) packages in R.

### 4.6. Quantitative RT-PCR Analysis (qRT-PCR)

The total RNA was extracted using TRIzol^®^ RNA Isolation Reagents (Invitrogen™, Waltham, Massachusetts, USA), and the DNA was cleaved using DNase I kit (Cat. No. 18068015, Invitrogen, USA). The first strand cDNA was synthesized using the cDNA Synthesis Kit (Cat. no. 6210A, Takara, Japan) and then used as template for qRT-PCR reactions. The LightCycler 480 System (Roche, Penzberg, Upper Bavaria, Germany) was used for conducting the qRT-PCR. The *GmUKN1* (*Glyma.12G020500*) [59] was used as the internal control to quantify the relative expression level of target genes. All primers for qRT-PCR are listed in Table S5. Reactions were performed using the SYBR Premix Ex Taq kit (Cat. no. RR420A, Takara, Kusatsu, Shiga, Japan) following the manufacturer’s protocol. The PCR amplification conditions were set as the following: 95 °C 10 min, 40 cycles of 15 s at 95 °C, and 1 min at 60 °C, the melting curve analysis was executed to verify the specificity of the primers with the following stages: 95 °C for 15 s, 60 °C for 1 min, and 95 °C for 15 s. Each sample was repeated three times.

### 4.7. Pathway and GO Enrichment Analysis and MapMan Metablic Map

The KEGG pathway enrichment analysis was performed using the clusterProfiler (Version 3.8.1) [60]. The *p* value less than 0.01 was regarded as significantly enriched. The agriGO was used for GO enrichment analysis [61]. 0.01 was set as the threshold for *FDR* value. The open source MapMan software [62] was used to generate the metabolic map of gene expression patterns in the fatty acid synthesis pathway.

### 4.8. Co-Expression Network Analysis

After removing the genes with FPKM value less than 1 in all samples, 28072 genes were used for the gene co-expression network analysis by WGCNA (version 1.49) [15]. The network construction and consensus module detection were performed by applying TOM and DynamicTreeCut functions. After exploring the soft thresholds, we finally set the power *β* to 26 (Figure S4), and minimum module size as 150. The correlation between genes was measured by Pearson’s correlation and the co-expressed gene sets (modules) were detected using hierarchical clustering method. The Pearson correlation between oil content and gene expression data was calculated as the gene significance (GS) value. The expression pattern analysis of the modules was conducted by STEM [63]. The visual network was constructed using Cytoscape (Version 3.6.1) [64].

### 4.9. Heatmap Analysis

Heatmap and cluster analysis of the candidate genes was performed using MEV (version 4.9) [65] via the hierarchical clustering method.

## 5. Conclusions

In this study, we investigated the dynamic changes in oil content and transcriptome in the developing seeds of soybean variety NN1138-2. Results showed that the up-regulated genes at the critical stage of seed oil accumulation are enriched in carbohydrate metabolism, glycolysis metabolism, photosynthesis, oxidation reduction and fatty acid metabolism, which suggests the transcriptional changes of genes in these pathways may have a positive impact on the physiological changes and oil content in soybean seeds. Furthermore, six gene co-expression modules and 124 key candidate DEGs were identified related to the oil content in developing soybean seeds. This study combined phenotypic changes, differential expression and co-expression analysis to reveal the molecular basis of soybean seed oil synthesis, which lay a foundation to further understand the seed oil accumulation process and provide candidate genes for molecular breeding to improve soybean seed oil content.

## Supplementary Materials

The following are available online at https://www.mdpi.com/1422-0067/20/9/2202/s1, Figure S1. Verification of RNA-Seq results by qRT-PCR. Comparison of RNA sequencing (RNA-Seq) data (blue bar) with qRT-PCR data (orange line). The normalized expression levels (FPKM) from the RNA-Seq results are indicated on the y-axis to the left. The relative qRT-PCR expression level is shown on the y-axis to the right. *UKN1* (*Glyma.12G020500*) was used as an internal control. Error bars represent the standard errors of means across three replications. Figure S2. Expression pattern analysis of the 12 modules detected by weighted correlation network analysis (WGCNA). Figure S3. Heatmap showing the expression patterns of candidate genes related to soybean seed oil synthesis at different developmental stages. Each cell was colored based on the log2(FPKM+1), and the darker red represent higher expression values, the darker green represent lower expression values. Figure S4. Threshold power (*β*) value determination from WGCNA output. (A) shows the scale free topology index in y-axis as a function of the soft threshold in x-axis. The red line indicates when the *β* value = 26, square of correlation coefficient (*R*^2^) is equal to 0.9 (B) shows the mean connectivity in y-axis reaching a saturation point at threshold *β* value = *26* in x-axis. Table S1. Top 10 DEGs in NN1138-2 seeds comparing 20 DAF with 10 DAF. Table S2. Functional enrichment analysis of DEGs comparing 20 DAF with 10 DAF in NN1138-2 seeds. Table S3. Top 10 hub genes in each of the six modules showing significant correlation with oil content. Table S4. The 124 candidate genes related to oil accumulation in soybean seeds. Table S5. Primers used for qRT-PCR in this study.

## Abbreviations

ABI3	Abscisic acid-insensitive 3
ACCase	Acetyl-CoA carboxylase
BP	Biology process
BC	Biotin carboxylase subunit
BCCP	Biotin carboxyl carrier protein
DAF	Days after flowering
DAG	Diacylglycerol
DGK	Diacylglycerol kinase
DEG	Differently expressed gene
DW	Dry weight
EAR	Enoyl-ACP reductase
FDR	False discovery rate
FAD2	Fatty acid desaturase 2
FAD3	Fatty acid desaturase 3
FPKM	Fragments per kilobase per million
GS	Gene significance
HAD	Hydroxyacyl-ACP dehydrase
HSD	11-β-Hydroxysteroid dehydrogenase-like
KAR	Ketoacyl-ACP reductase
KAS I	Ketoacyl-ACP synthase I
KAS II	Ketoacyl-ACP synthase II
KAS III	Ketoacyl-ACP synthase III
MCMT	Malonyl-CoA: ACP malonyltransferase
ME	Module eigengene
MM	Module membership
PDAT	Phospholipid: diacylglycerol acyltransferase
RNA-Seq	RNA sequencing
TAGs	Triacylglycerols
WGCNA	Weighted correlation network analysis
