# Supplementary figures and images for: Dynamic Transcriptome Changes Related to Oil Accumulation in Developing Soybean Seeds
Source: Int J Mol Sci. 2019 May 5;20(9):2202. doi: 10.3390/ijms20092202 (PMC6539092; doi:10.3390/ijms20092202)

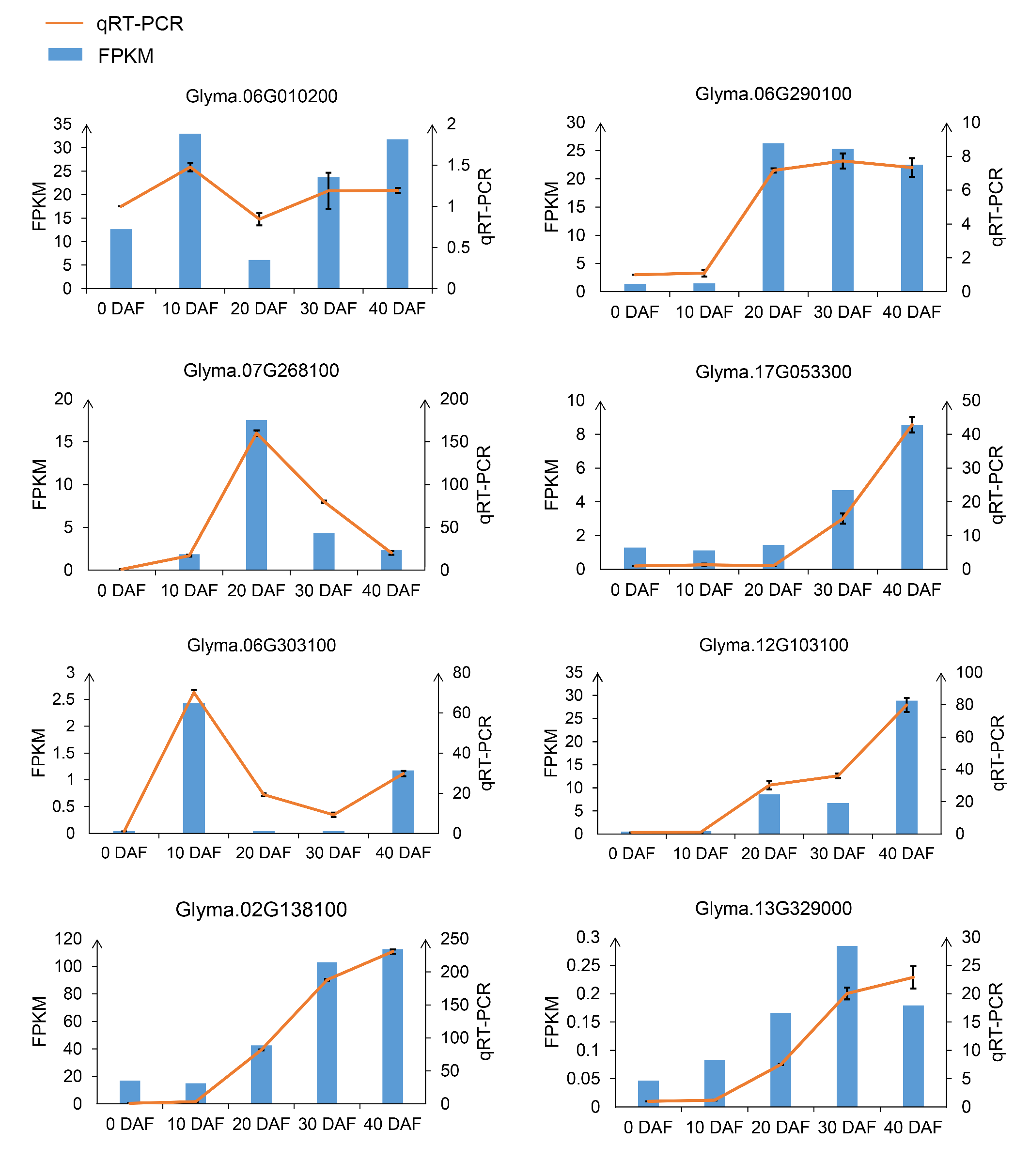

Supplement: Supplementary file 1 [file ijms-20-02202-s001.zip › Figure S1.tif]

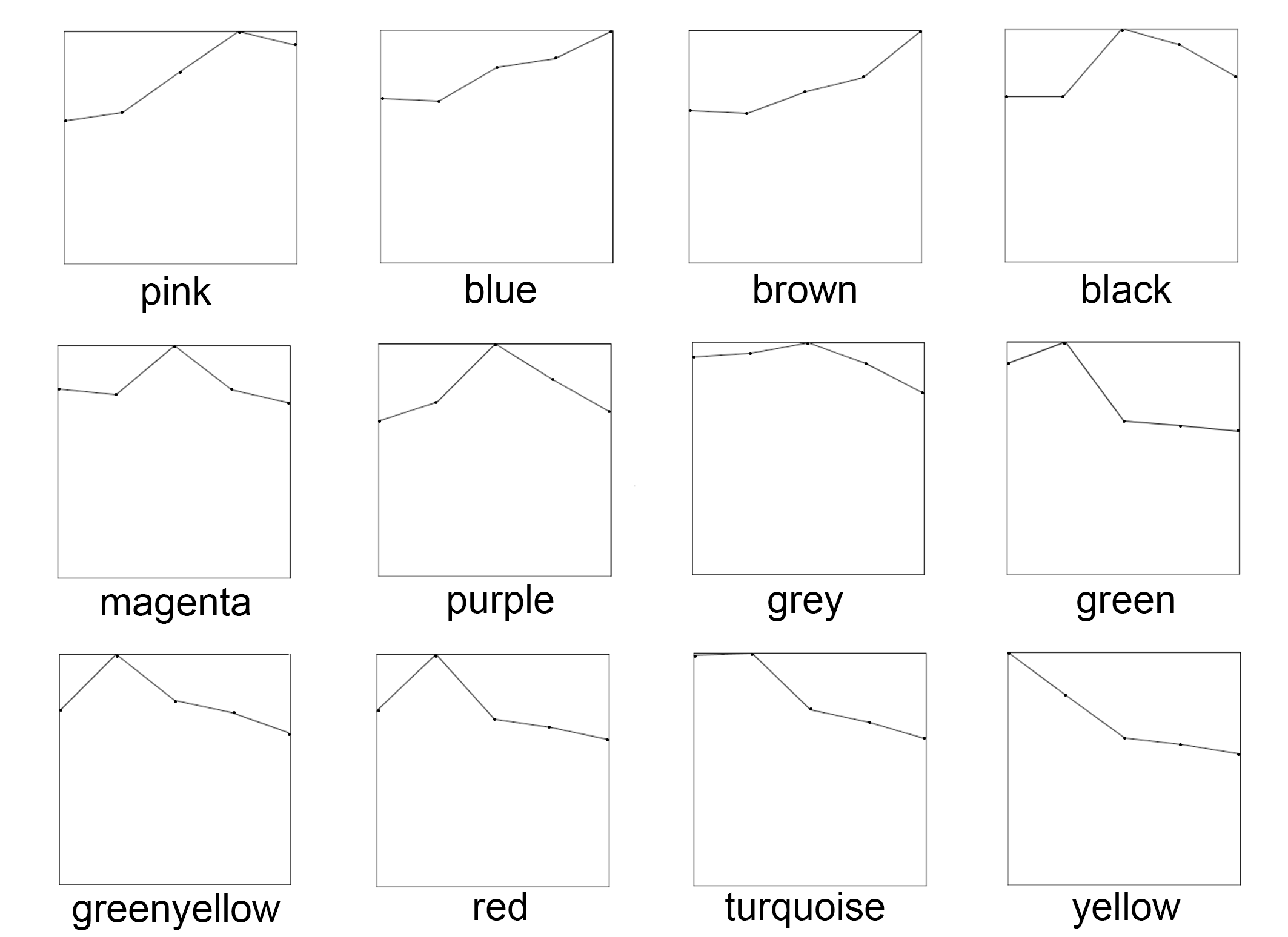

Supplement: Supplementary file 1 [file ijms-20-02202-s001.zip › Figure S2.tif]

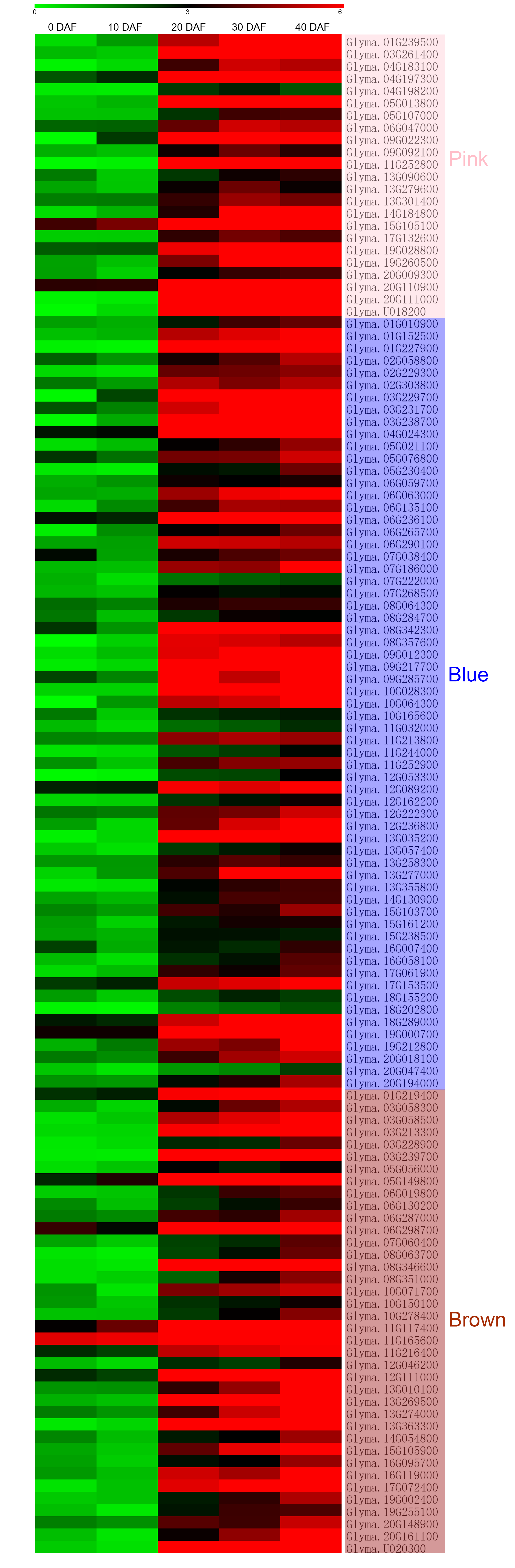

Supplement: Supplementary file 1 [file ijms-20-02202-s001.zip › Figure S3.tif]

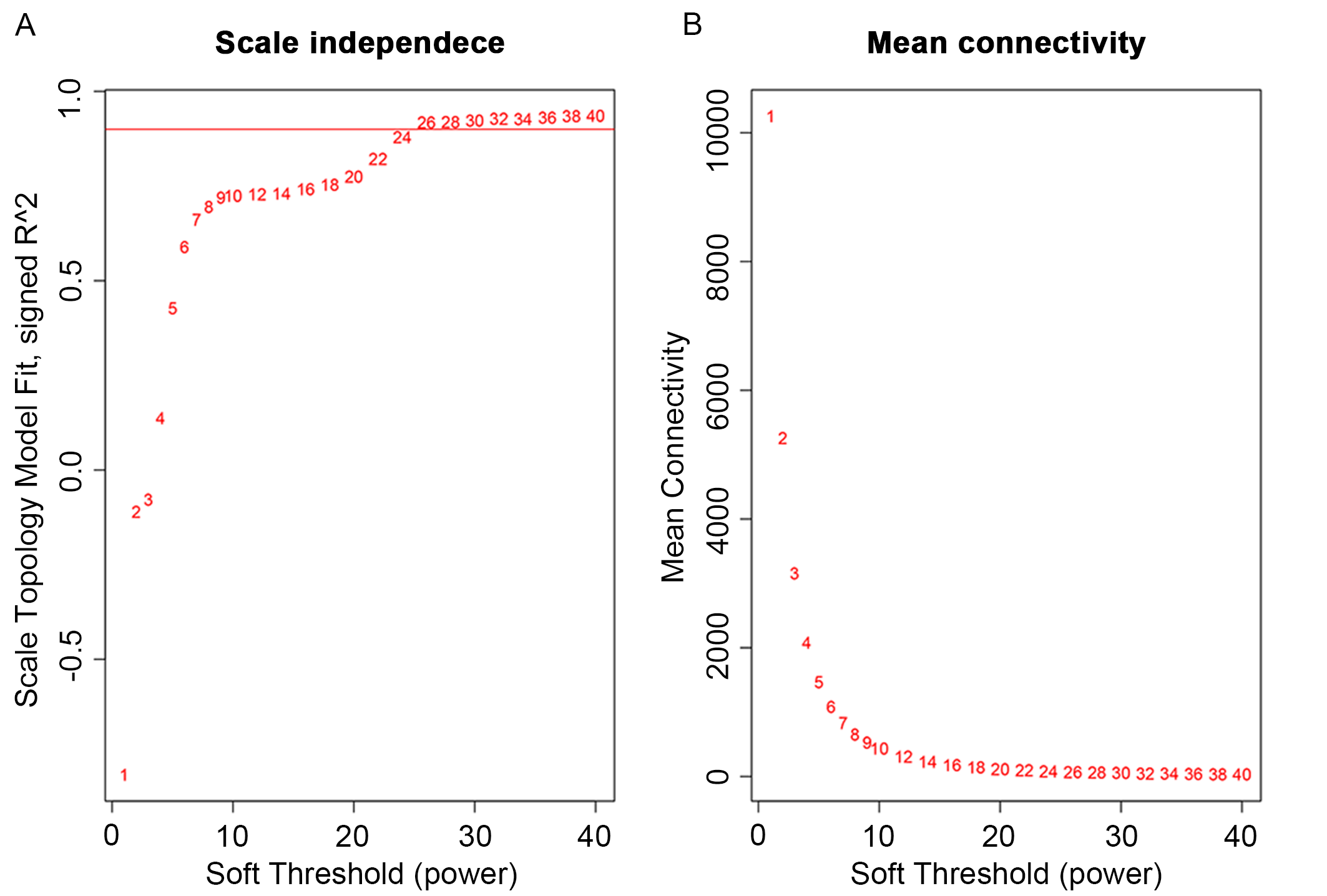

Supplement: Supplementary file 1 [file ijms-20-02202-s001.zip › Figure S4.tif]
